# Supplementary material for: Androgen signaling uses a writer and a reader of ADP-ribosylation to regulate protein complex assembly
Source: Nat Commun. 2021 May 11;12:2705. doi: 10.1038/s41467-021-23055-6 (PMC8113490; doi:10.1038/s41467-021-23055-6)
Supplement: Supplementary file 1 — Supplementary Information [file 41467_2021_23055_MOESM1_ESM.pdf]

Supplementary Information

Androgen signaling uses a writer and a reader of ADP-ribosylation to regulate protein complex assembly

C.S. Yang, K. Jividen, T. Kamata, N. Dworak, L. Oostdyk, B. Remlein, Y. Pourfarjam, I.-K. Kim, K.P. Du, T. Abbas, N. E. Sherman, D. Wotton, and B. M. Paschal

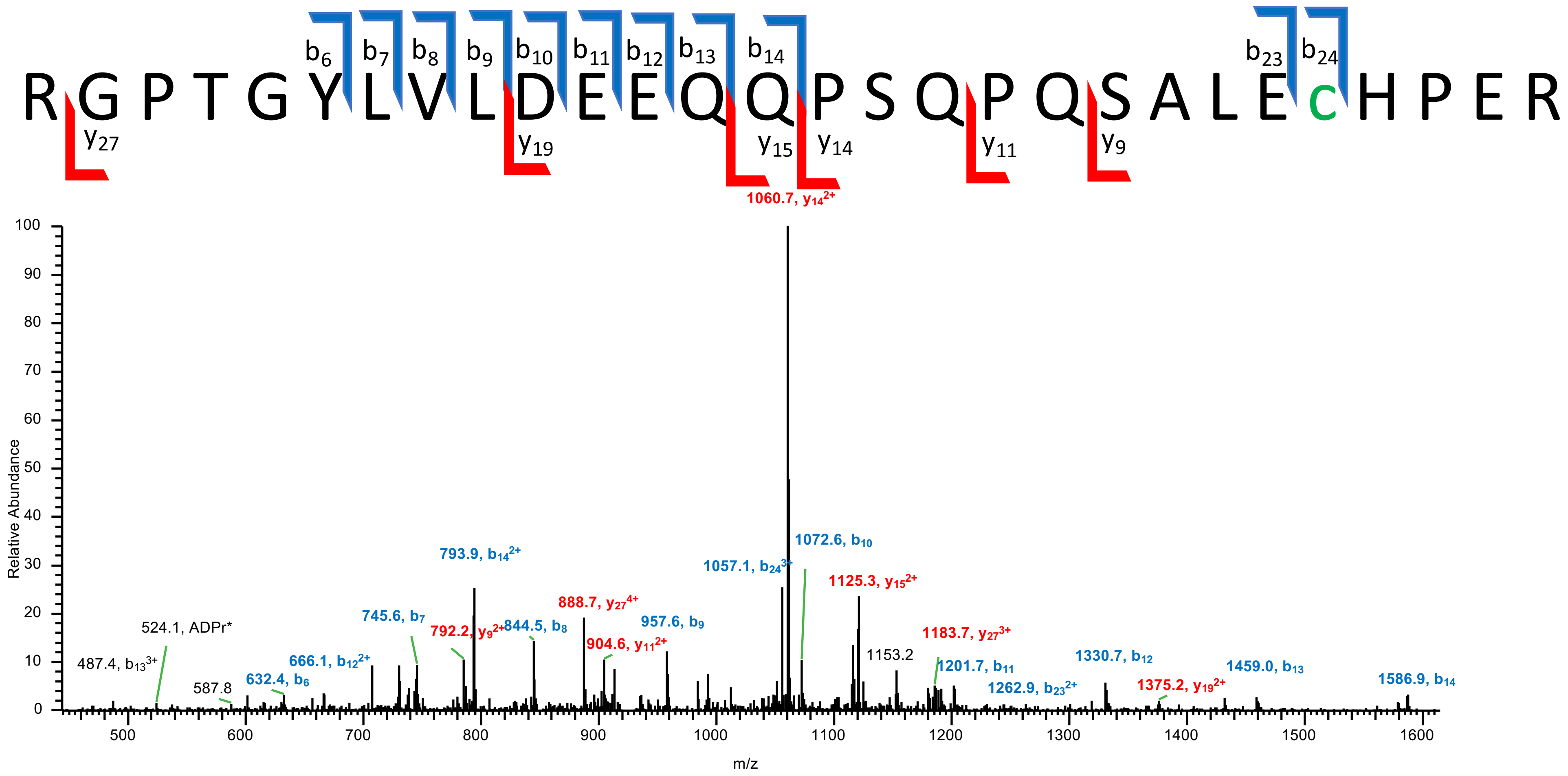

Supplementary Fig. 1. MS/MS spectra from CID of ADP-ribosylated peptide (C125) from AR

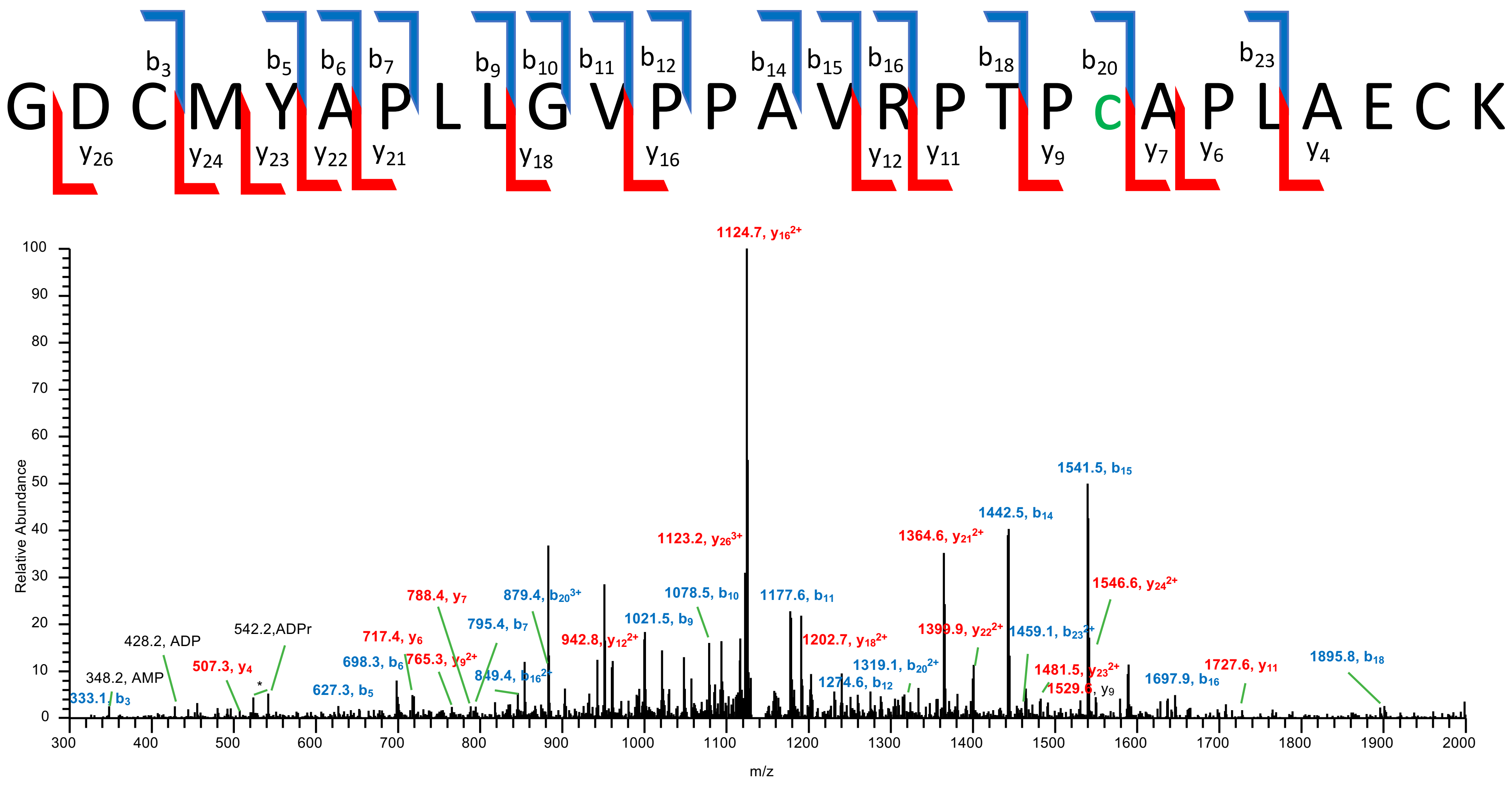

Supplementary Fig. 2. MS/MS spectra from CID of ADP-ribosylated peptide (C284) from AR

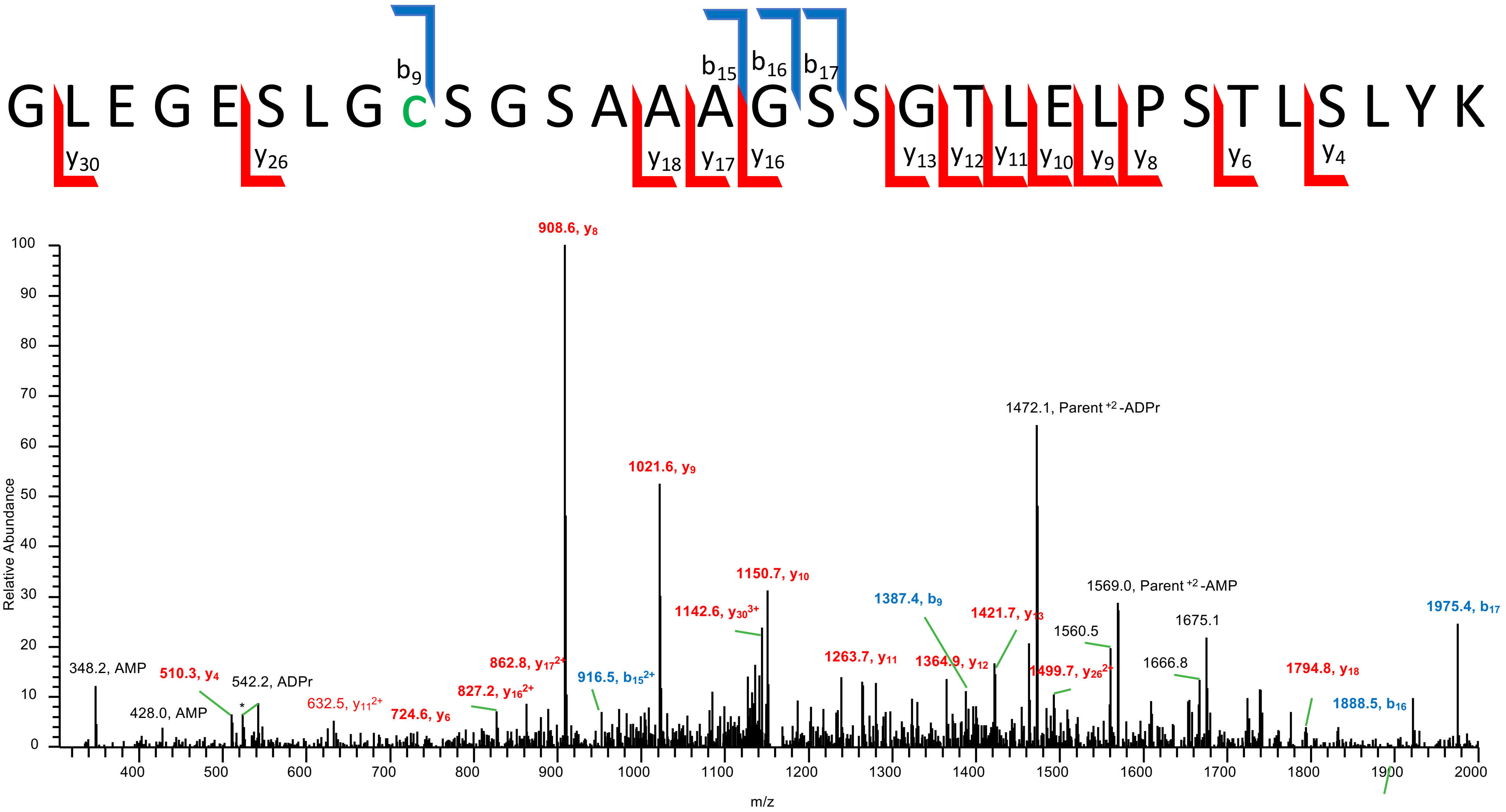

Supplementary Fig. 3. MS/MS spectra from CID of ADP-ribosylated peptide (C327) from AR

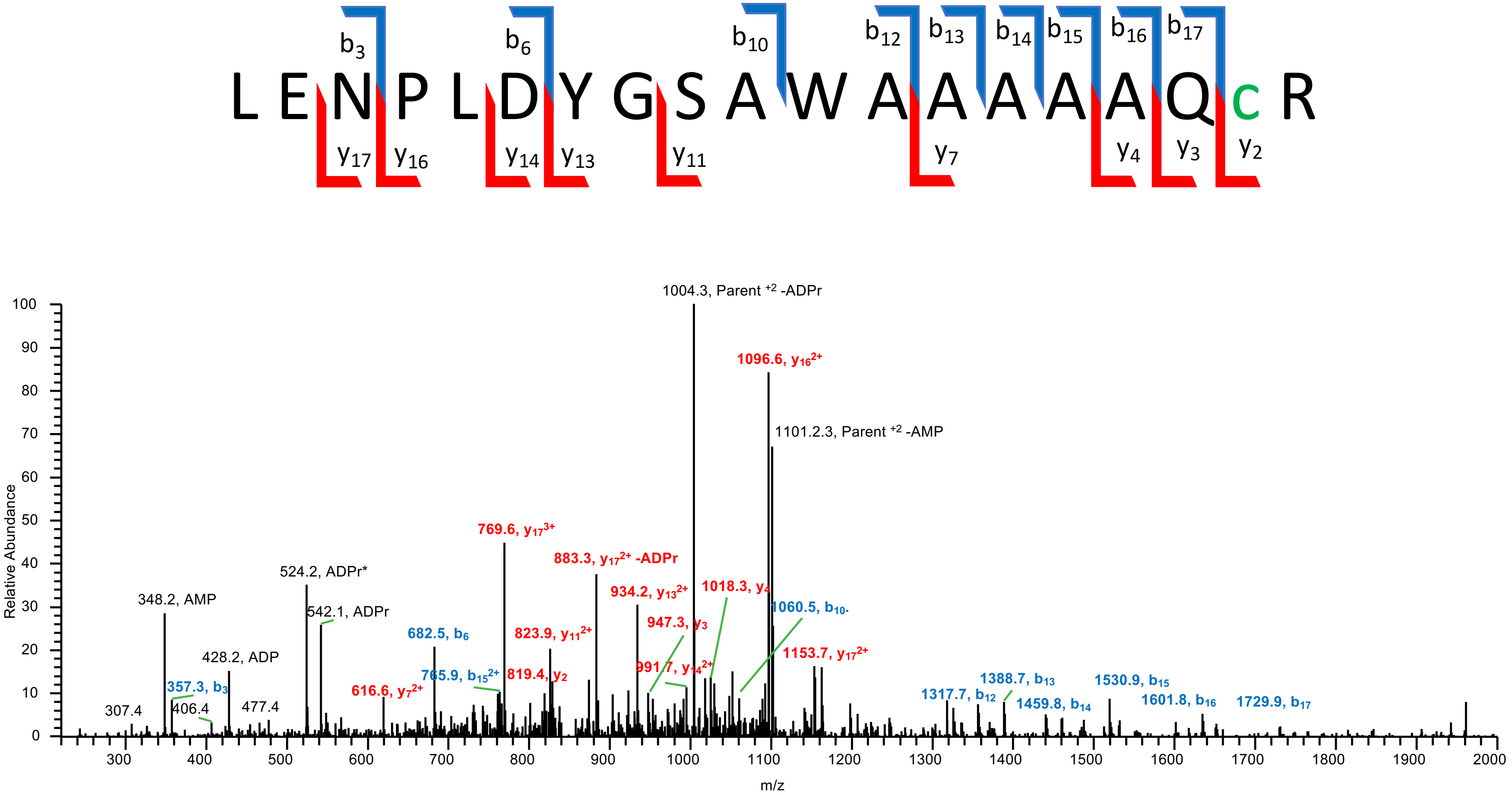

Supplementary Fig. 4. MS/MS spectra from CID of ADP-ribosylated peptide (C406) from AR

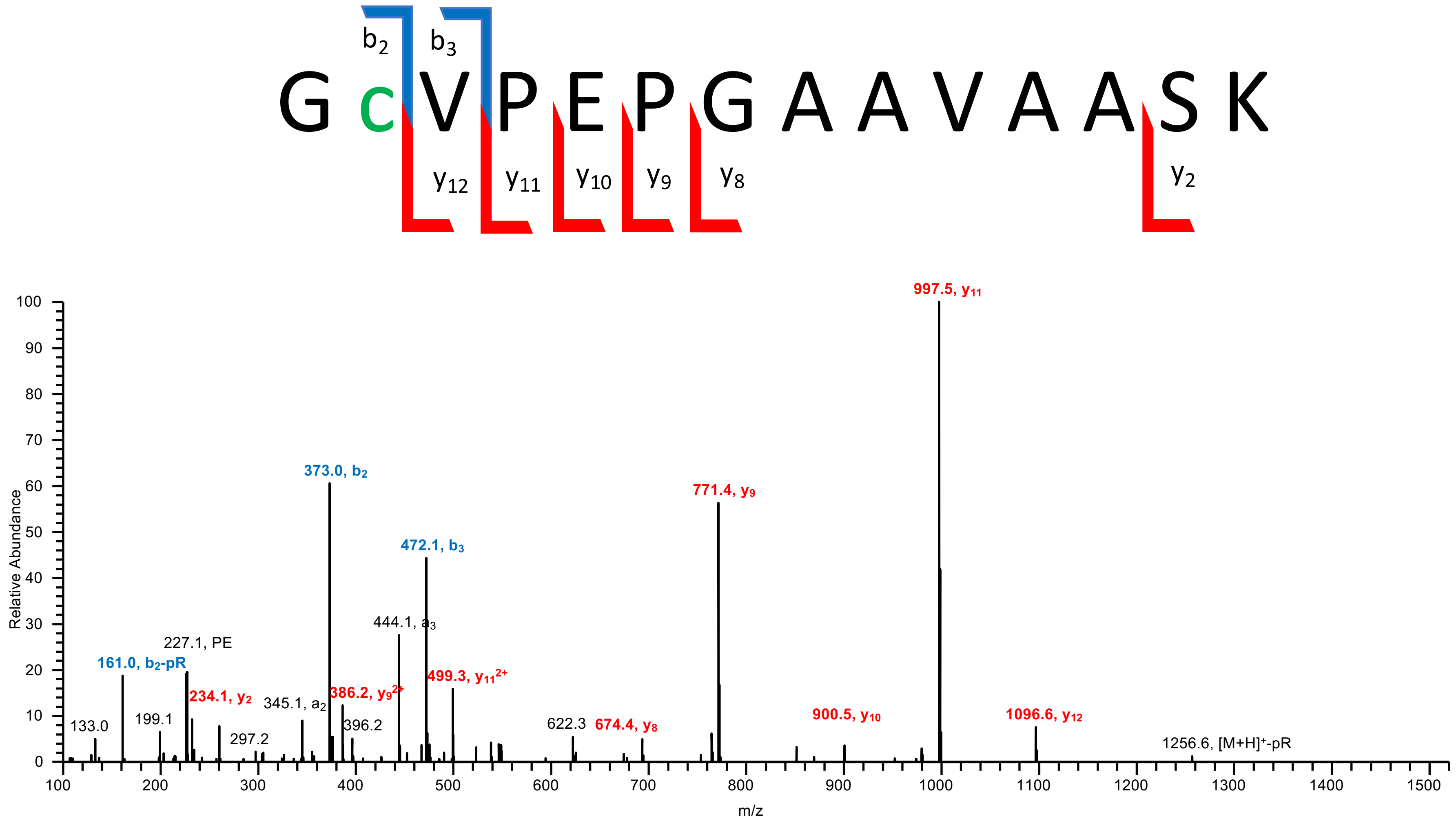

Supplementary Fig. 5. MS/MS spectra from CID of phospho-ribosylated peptide (C131) generated by NUDT16 treatment of ADP-ribosylated AR

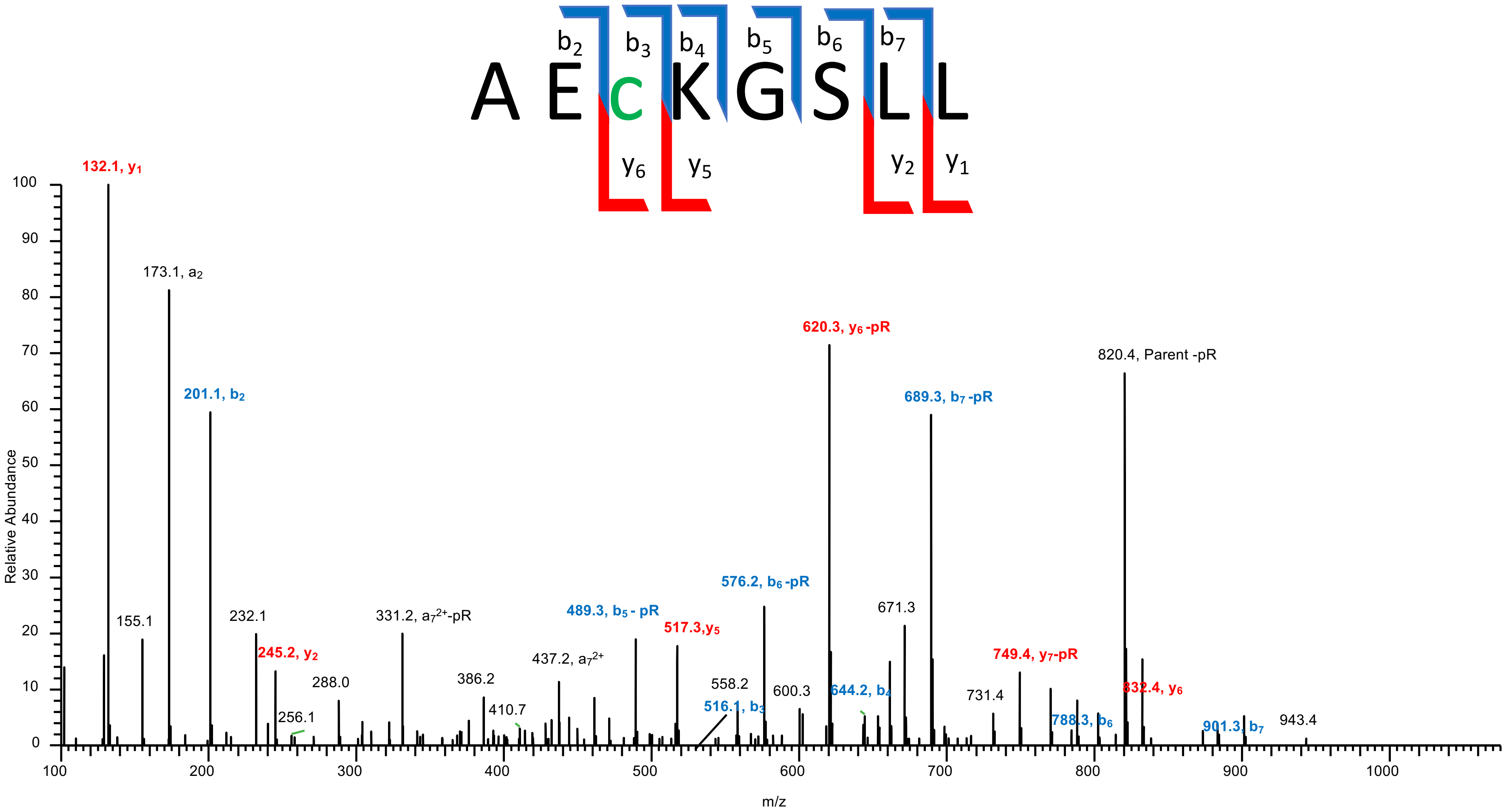

Supplementary Fig. 6. MS/MS spectra from CID of phospho-ribosylated peptide (C290) generated by NUDT16 treatment of ADP-ribosylated AR

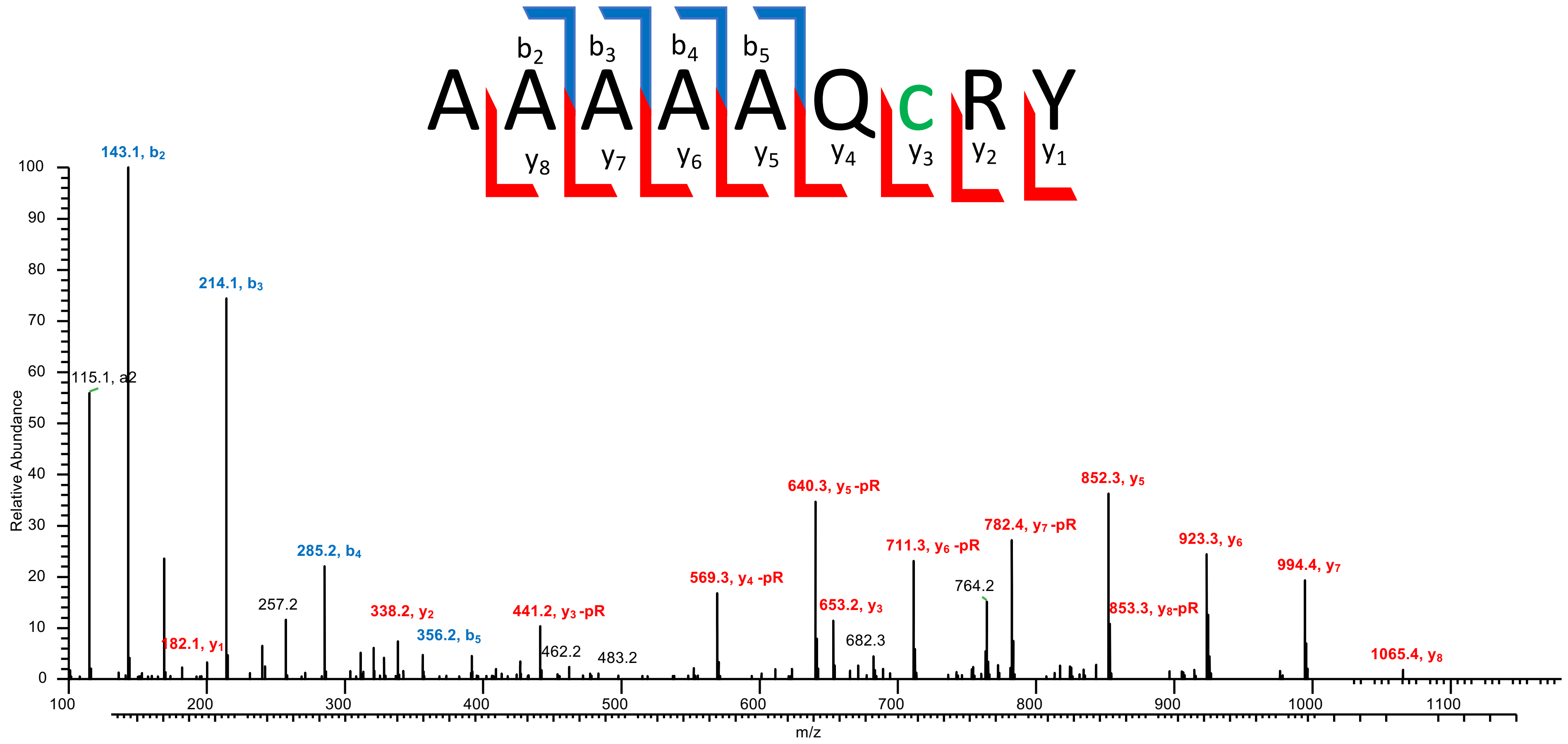

Supplementary Fig. 7. MS/MS spectra from CID of phospho-ribosylated peptide (C406) generated by NUDT16 treatment of ADP-ribosylated AR

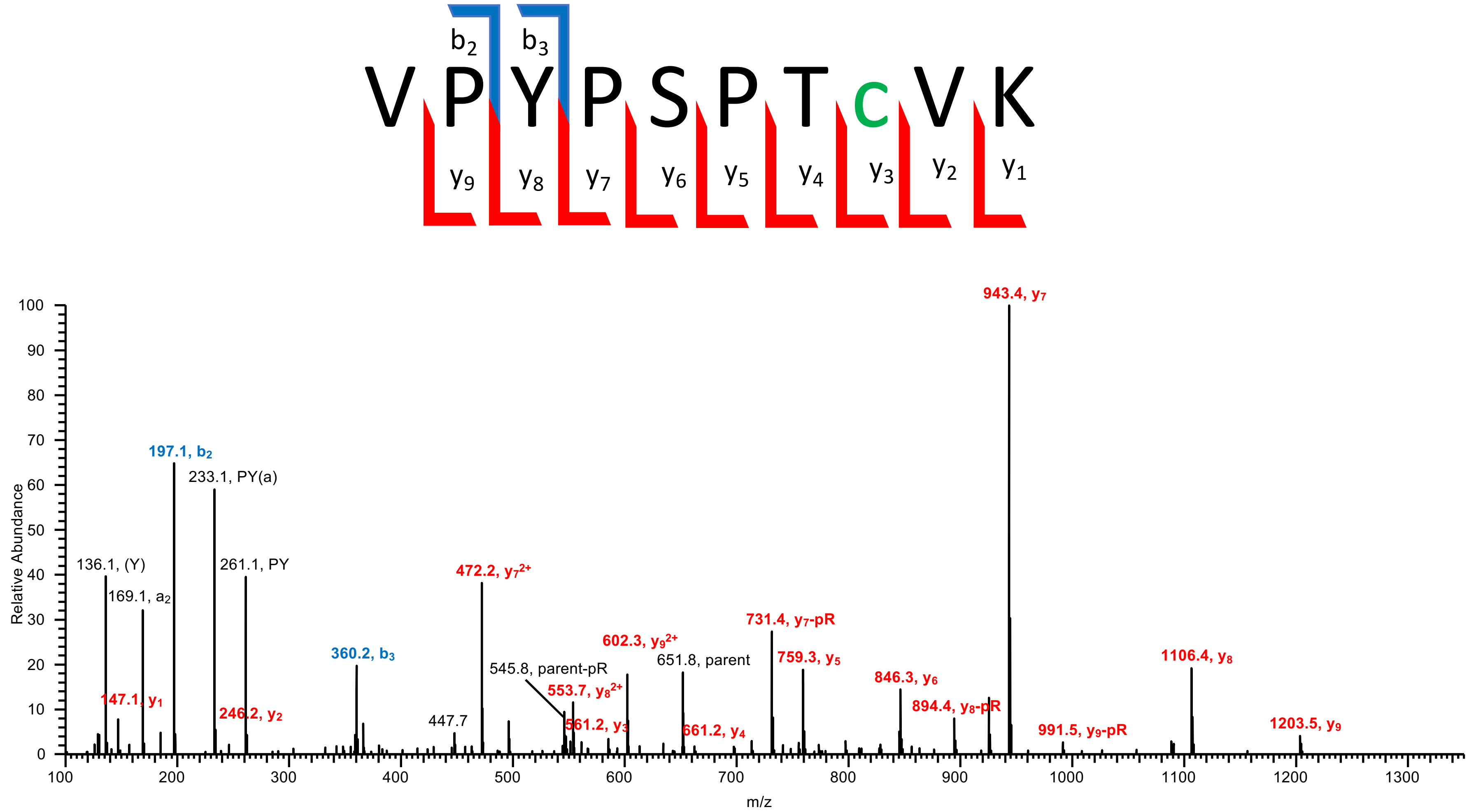

Supplementary Fig. 8. MS/MS spectra from CID of phospho-ribosylated peptide (C519) generated by NUDT16 treatment of ADP-ribosylated AR

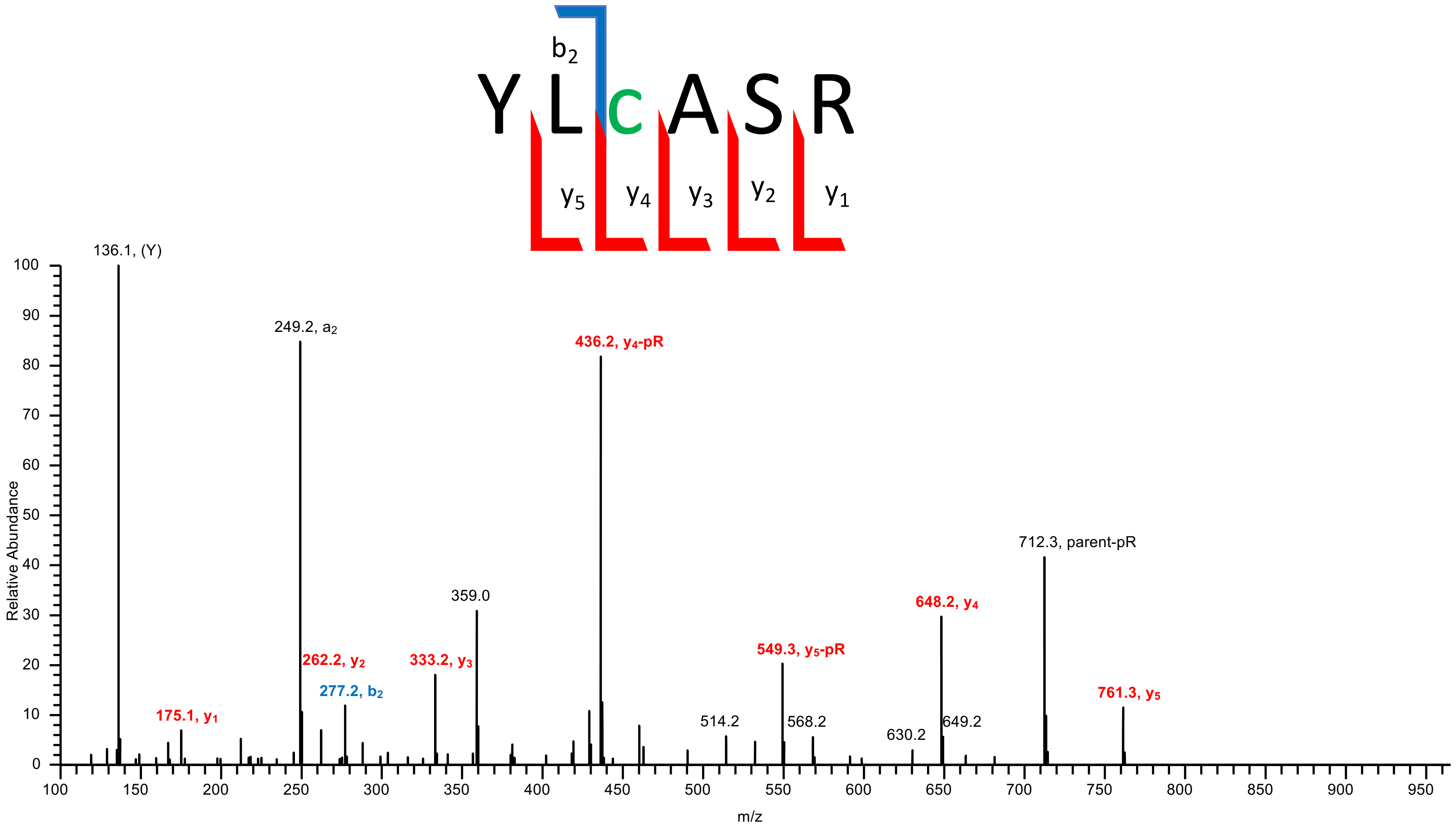

Supplementary Fig. 9. MS/MS spectra from CID of phospho-ribosylated peptide (C596) generated by NUDT16 treatment of ADP-ribosylated AR

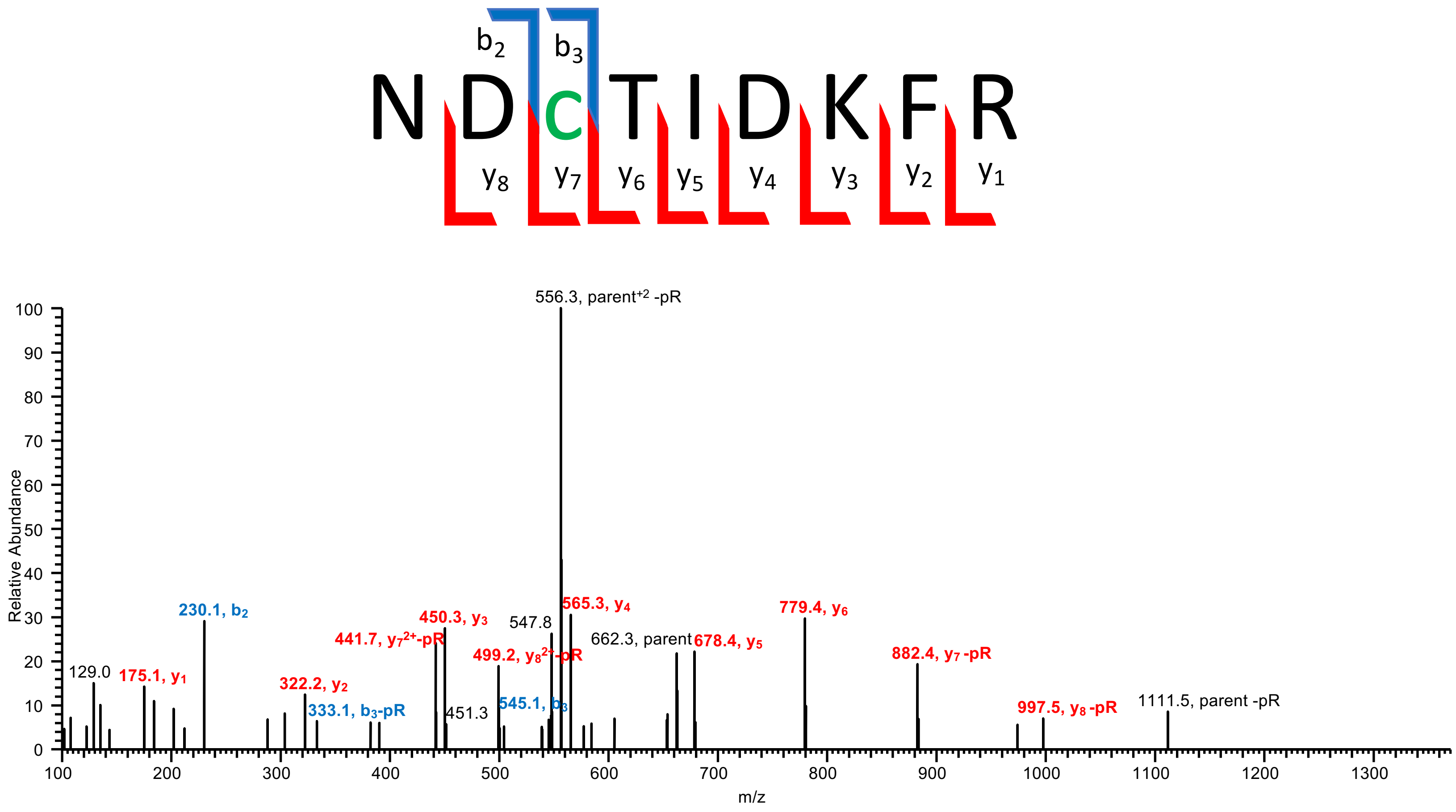

Supplementary Fig. 10. MS/MS spectra from CID of phospho-ribosylated peptide (C602) generated by NUDT16 treatment of ADP-ribosylated AR

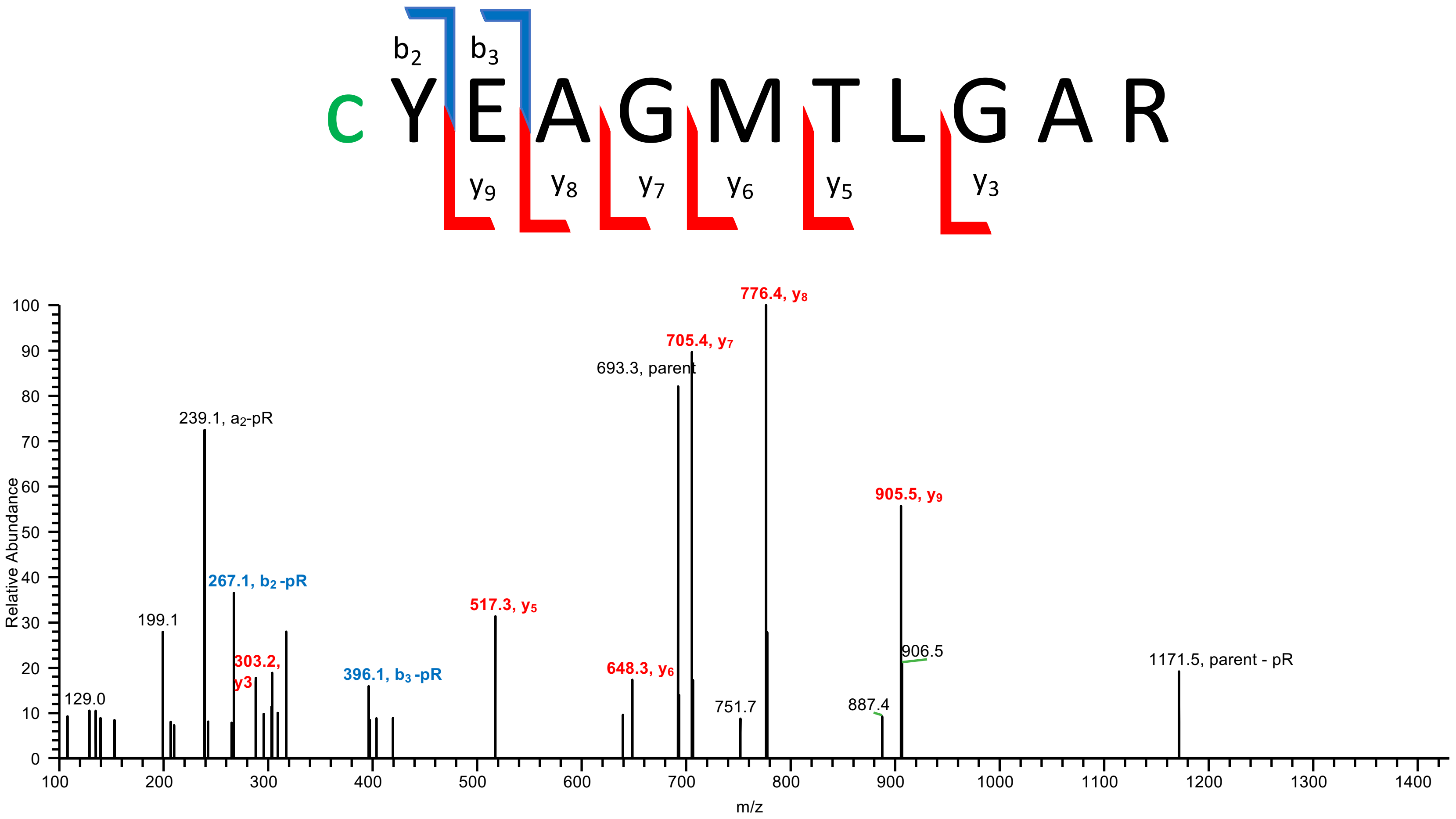

Supplementary Fig. 11. MS/MS spectra from CID of phospho-ribosylated peptide (C620) generated by NUDT16 treatment of ADP-ribosylated AR

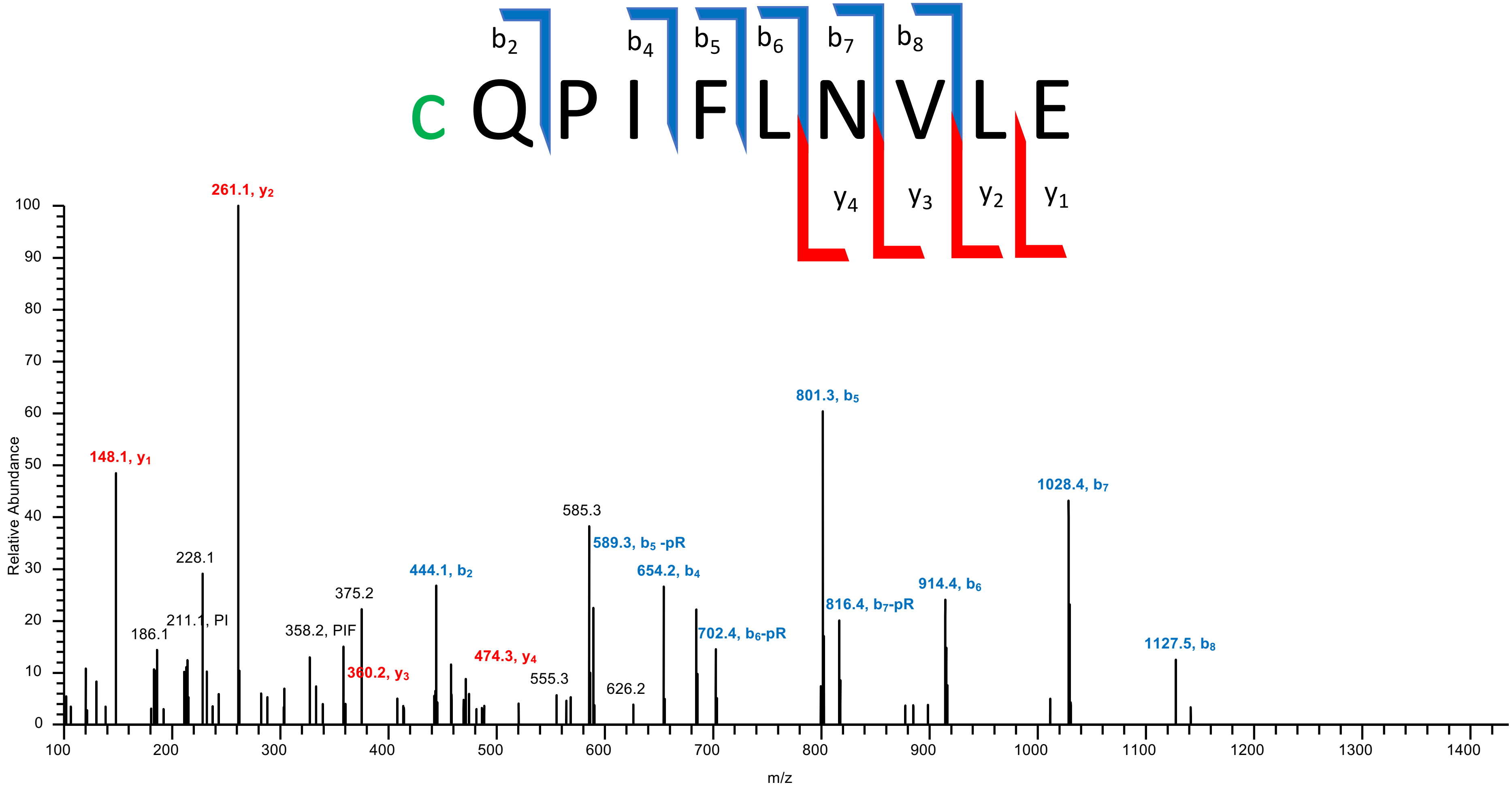

Supplementary Fig. 12. MS/MS spectra from CID of phospho-ribosylated peptide (C670) generated by NUDT16 treatment of ADP-ribosylated AR

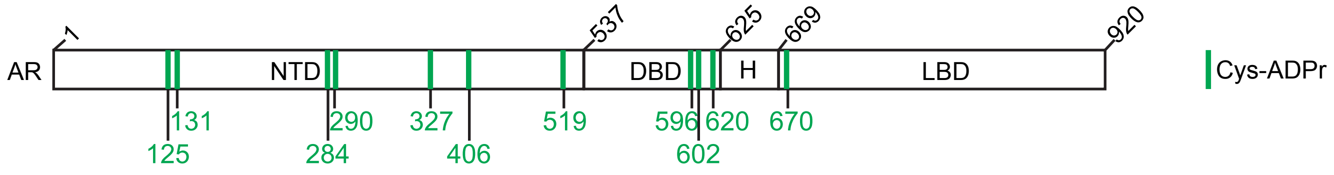

Supplementary Fig. 13. Diagram showing the eleven ADP-ribosylation sites (in green) in AR.

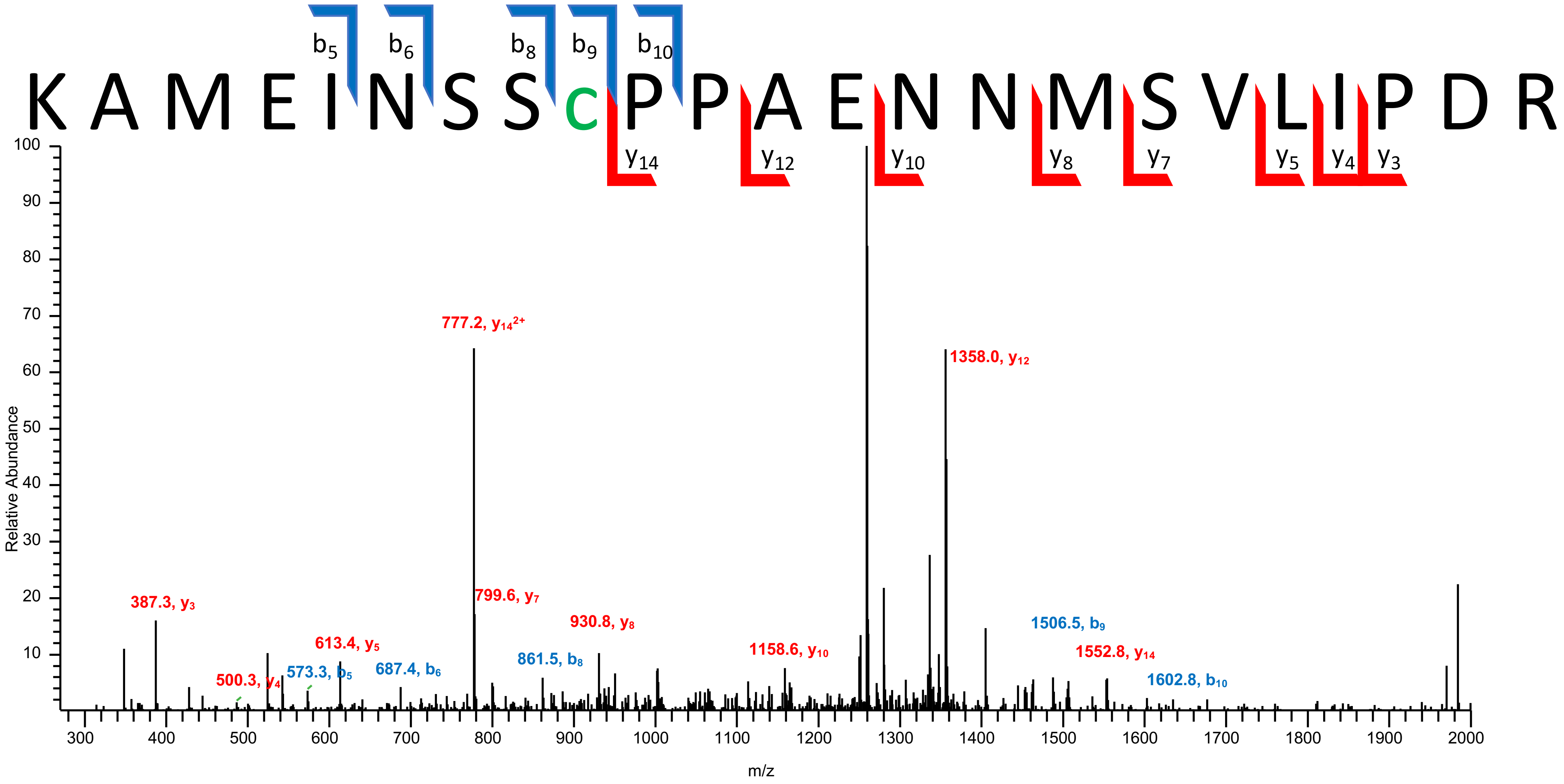

Supplementary Fig. 14. Mass spectra from CID of ADP-ribosylation peptide (C100) in human Parp7 purified from insect cells.

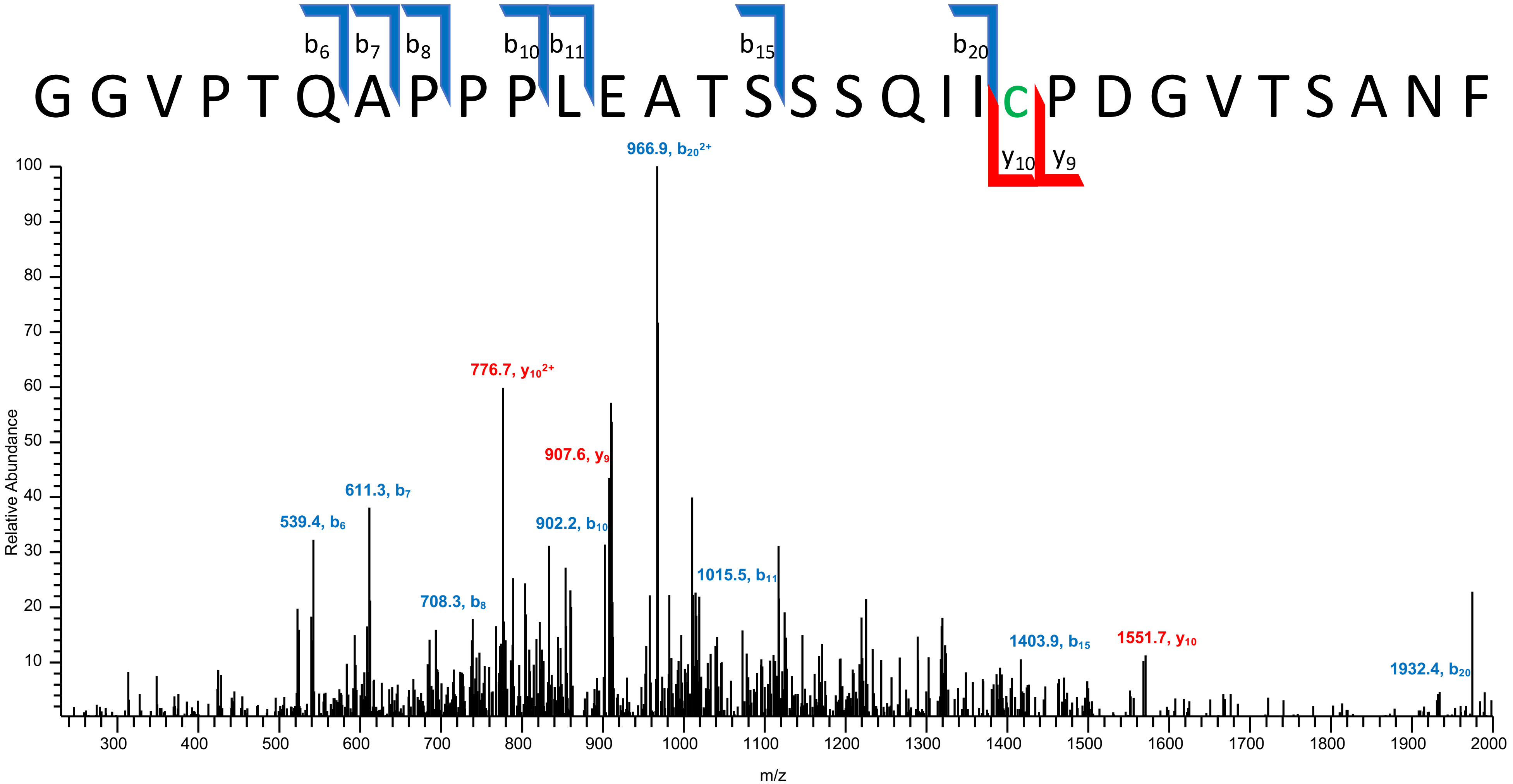

Supplementary Fig. 15. Mass spectra from CID of ADP-ribosylation peptide (C439) in human Parp7 purified from insect cells.

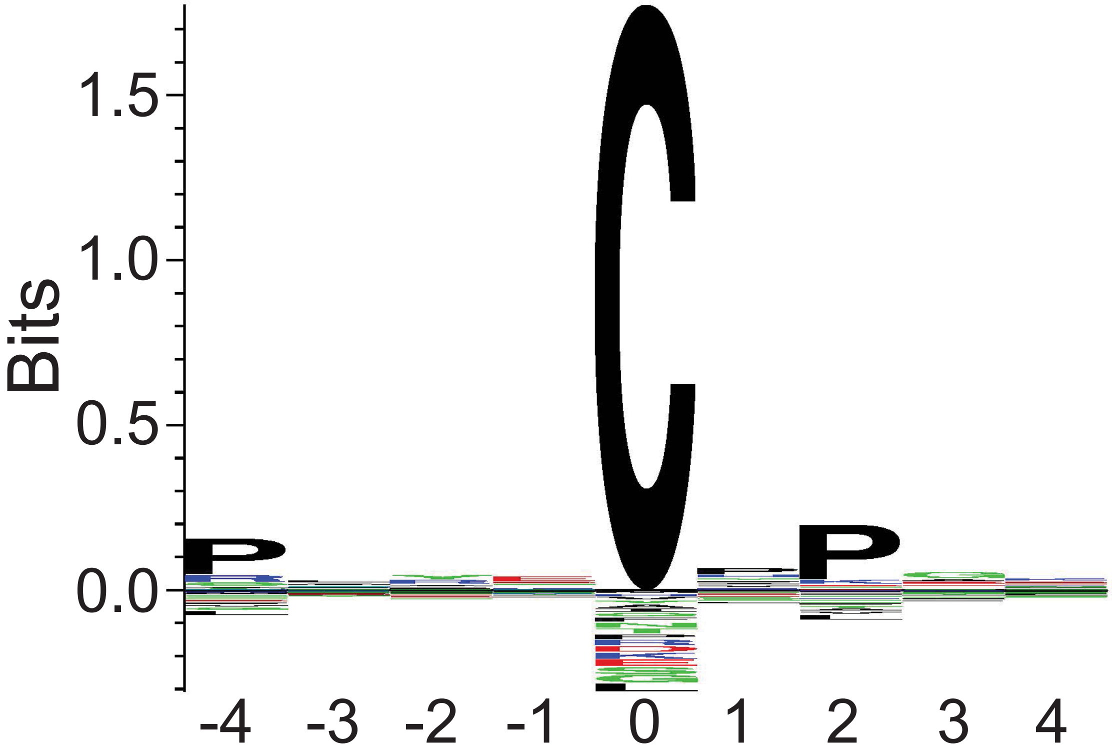

Supplementary Fig. 16. Visualization of AR and Parp7 ADP-ribosylation sites by Seq2Logo. Fourteen Cys ADP-ribosylation were used (11 sites from AR; 3 sites from Parp7). Each peptide sequence contained nine amino acids, with the acceptor Cys (designated as zero) flanked by four amino acids on the amino (-4, -3, -2, -1) and carboxyl (+1, +2. +3, +4) sides.The logo type was P-weighted Kullback-Leibler with a specificity threshold for clustering of 0.63.

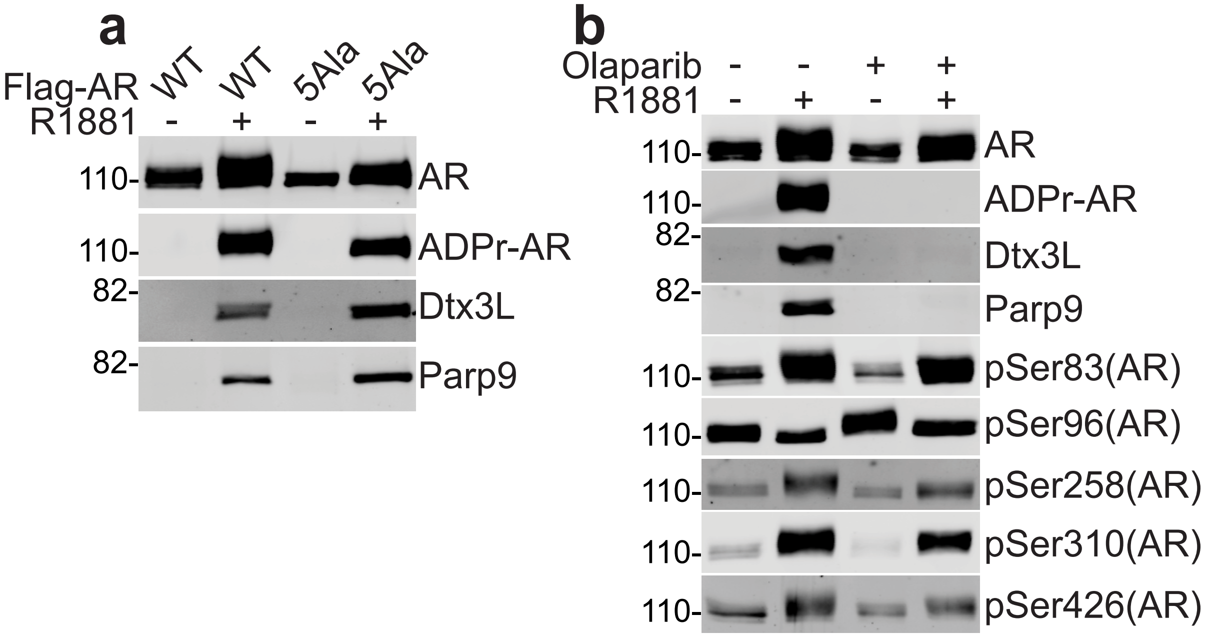

Supplementary Fig. 17. ADP-ribosylation and phosphorylation are indepentent AR modifications.
(**a**) Mutation of AR phosphorylation sites in the NTD (Ser83, 96, 258, 310, 426Ala; labeled 5Ala) does not inhibit androgen-induced ADP-ribosylation and complex formation with Dtx3L/Parp9. WT and mutant forms of AR were IP'd and blotted for ADP-ribosylation (Af1521 detection), Dtx3L, and Parp9.
(**e**) Inhibition of AR ADP-ribosylation with Olaparib does not preclude androgen induction of AR phosphorylation. PC3-AR/HA-Parp7 cells were co-treated with R1881 (2 nM) and Olaparib (10 μM) for 6 hrs, and IP'd AR was probed for ADP-ribosylation, complex formation, and phosphorylation with the indicated site-specific antibodies.
Source data are provided as a Source data file.

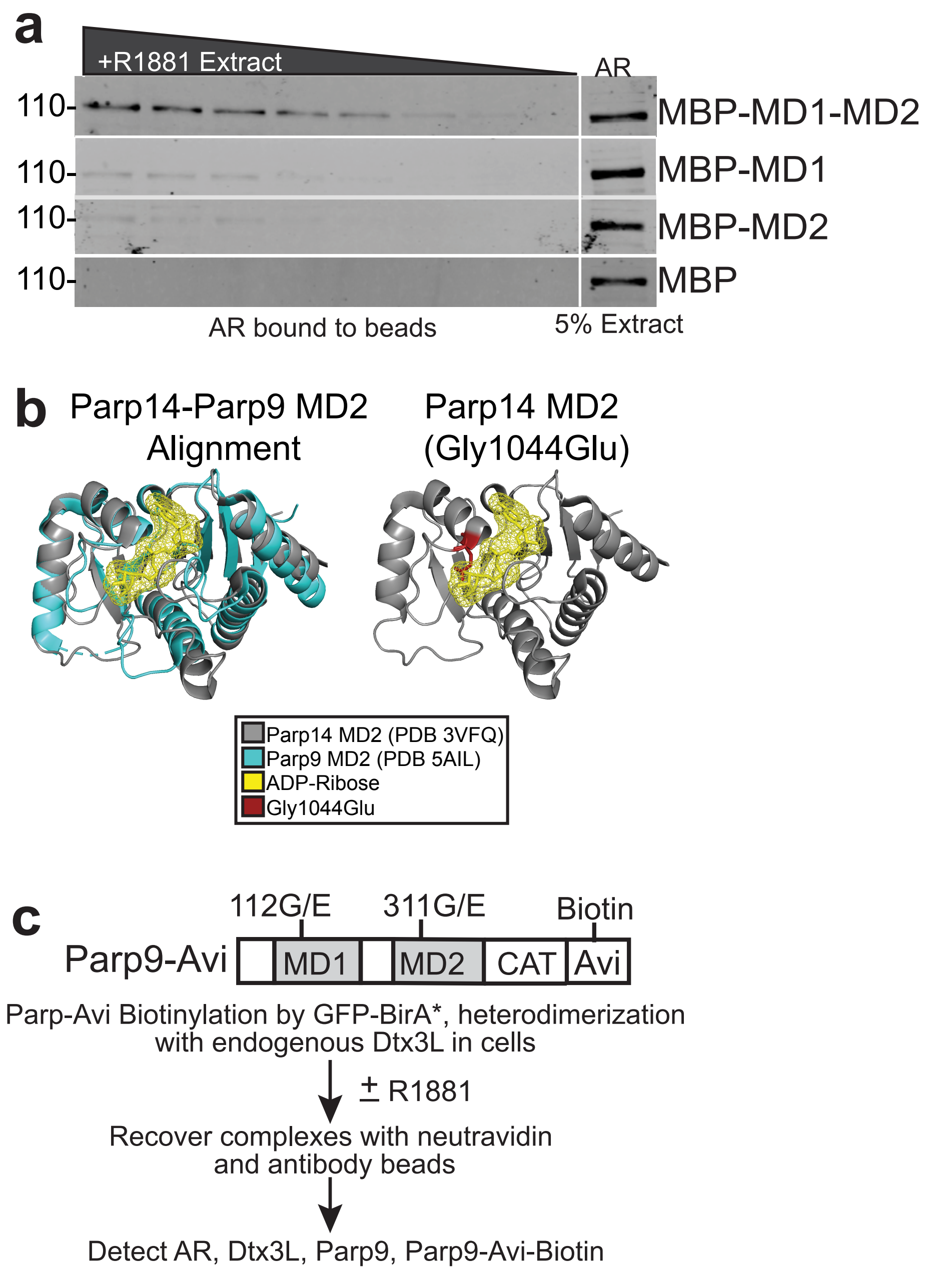

Supplementary Fig. 18. Parp9 macrodomain interactions with AR.
(a) Immunoblots showing AR binding to recombinant individual and tandem Parp9 macrodomains as a function of cell extract concentration. Source data are provided as a Source data file.
(b) Macrodomain structures from 3VFQ [https://www.rcsb.org/structure/3VFQ] (Parp14 MD2) and 5AIL [https://www.rcsb.org/structure/5AIL] (Parp9 MD2) aligned to show overall similarity, and the position of a conserved Gly in the ADP-ribose binding pocket that was mutated in Parp9. ADP-ribose (yellow mesh surface) and the binding pocket Gly-to-Glu (red wireframe) are depicted.
(c) Scheme for analyzing the Parp9 MD loss-of-function mutant.

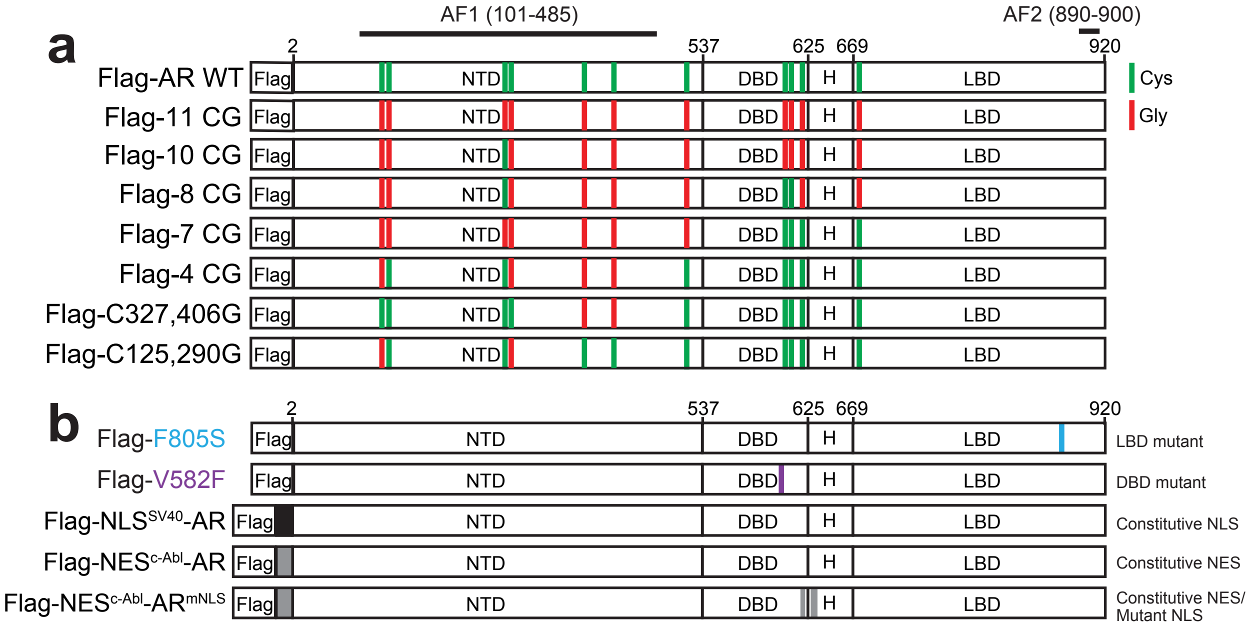

Supplementary Fig. 19. AR constructs used in the study.
(a) Diagrams of Flag-tagged AR WT, and Cys-Gly substitutions in the ADP-ribosylation sites identified by MS/MS.
(b) Diagrams of Flag-tagged AR mutants used to query the effects of mutations in the LBD (F805S) and the DBD (V582F), and effects on forced AR localization to the nucleus (NLS^{SV40}-AR) and the cytoplasm ($NES^{c\text{-}Abl}$-AR^{mNLS}).

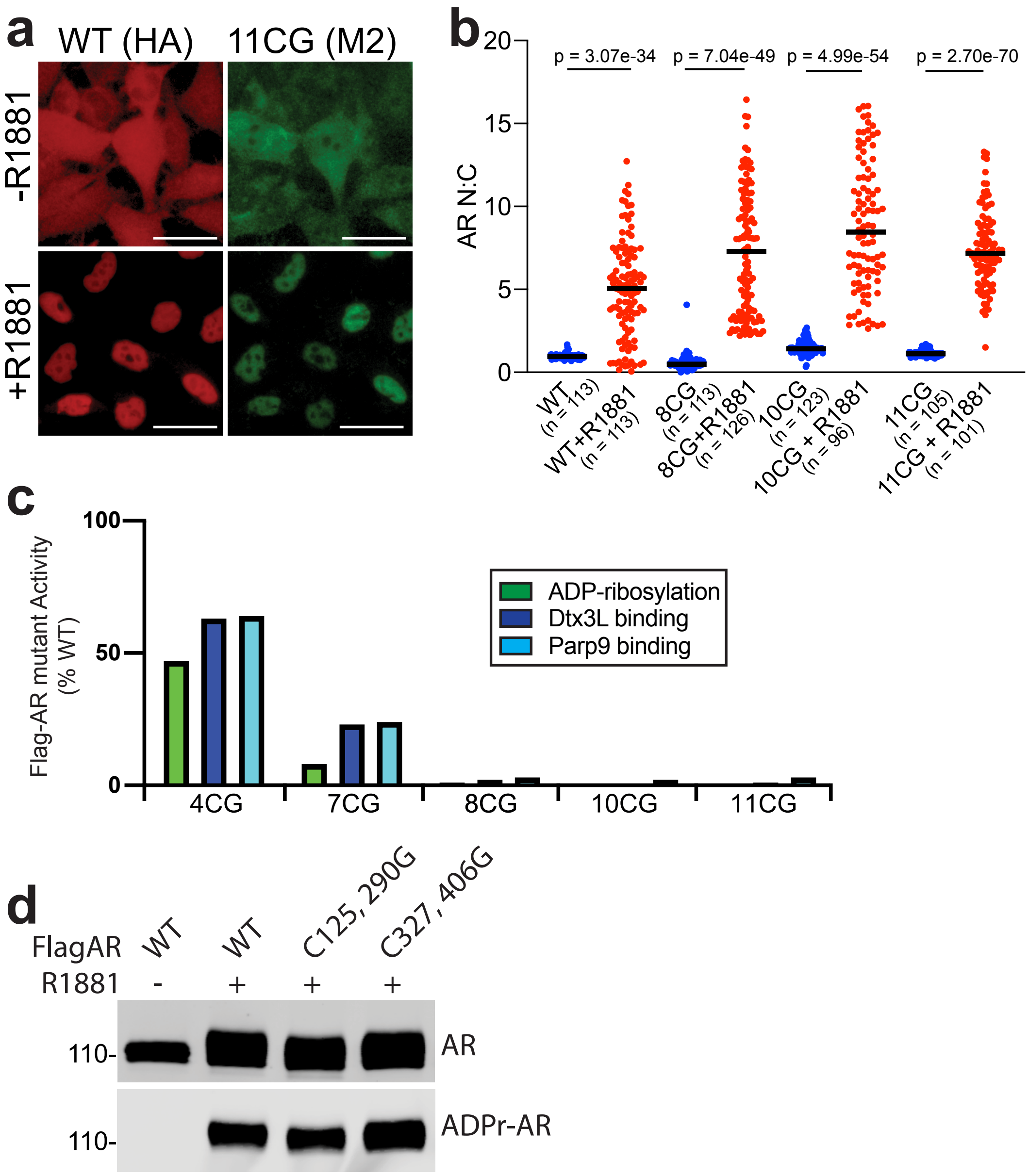

Supplementary Fig. 20. Characterization of AR ADP-ribosylation mutants.
(a) Immunofluorescence microscopy of co-expressed HA-tagged WT AR and Flag-tagged mutant (11 CG) demonstrating that both versions of AR undergo nuclear import in response to R1881. Scale bars = 20 microns.
(b) AR nuclear:cytoplasmic (N:C) values of Flag-tagged AR CG mutants and HA-AR WT expressed in PC3m cells. Each dot represents the AR N:C value from a single cell. The black lines represent medians.
(c) Compilation results of ADP-ribosylation, Dtx3L, and Parp9 binding to AR mutants plotted as a percentage of WT AR expressed in the PC3m(HA-Parp7) cells.
(d) AR ADP-ribosylation is maintained in 2CG mutants (C125,290G and C327,406G). Source data are provided as a Source data file.

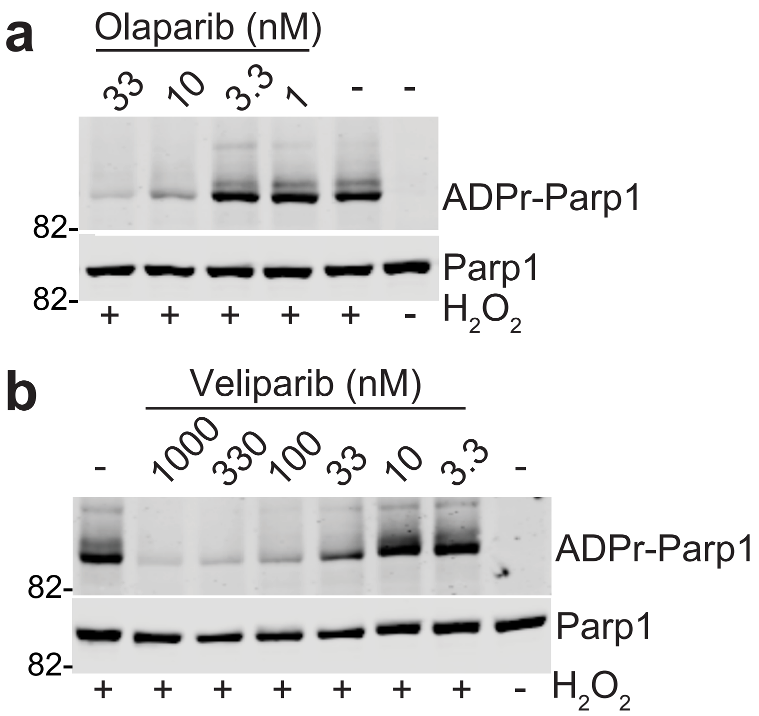

Supplementary Fig. 21. Sensitivity of Parp1 to Olaparib and Veliparib in prostate cancer cells.
(**a**) PC3-AR cells were treated with Olaparib (0-33 nM) for 30 min prior to exposure to H_2O_2 (0.88 mM) for 15 min. The samples were analyzed for ADP-ribose by Fl-Af1521 detection and Parp1 by immunoblotting. (**b**) PC3-AR cells were treated as described in Panel A except that Veliparib (0-1000 nM) was used.
Source data are provided as a Source data file.

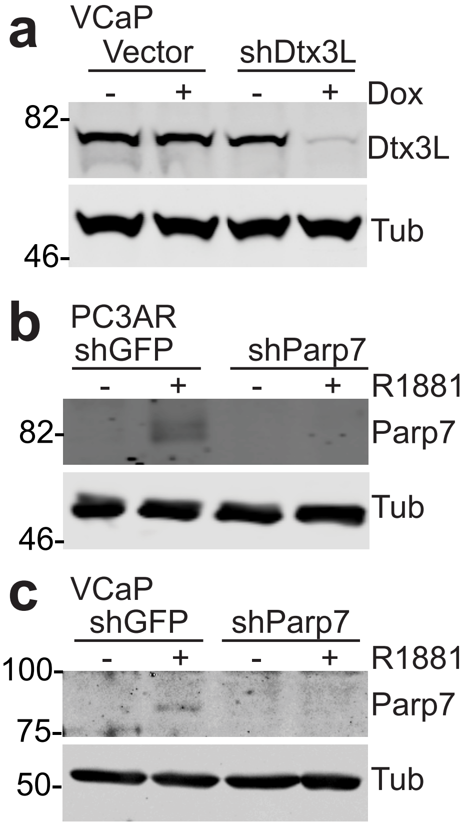

Supplementary Fig. 22. Validation of Dtx3L and Parp7 knockdowns.
(**a**) Dtx3L knockdown in VCaP cells using Dox-inducible shRNA (pLKO.Tet-On: vector control). Cells were treated with Doxycycline (Dox) for 4 days and the samples analyzed by immunoblotting for Dtx3L and Tubulin. (**b**) Stable knockdown of Parp7 in PC3-AR cells. shGFP and shParp7 PC3-AR cells were treated with R1881 overnight, and the samples analyzed by immunoblotting for Parp7 and Tubulin. (**c**) Stable knockdown of Parp7 in VCaP cells. shGFP and shParp7 VCaP cells were treated with R1881 overnight and MG132 (10 μM) for last hour, and the samples analyzed by immunoblotting for Parp7 and Tubulin.
Source data are provided as a Source data file.

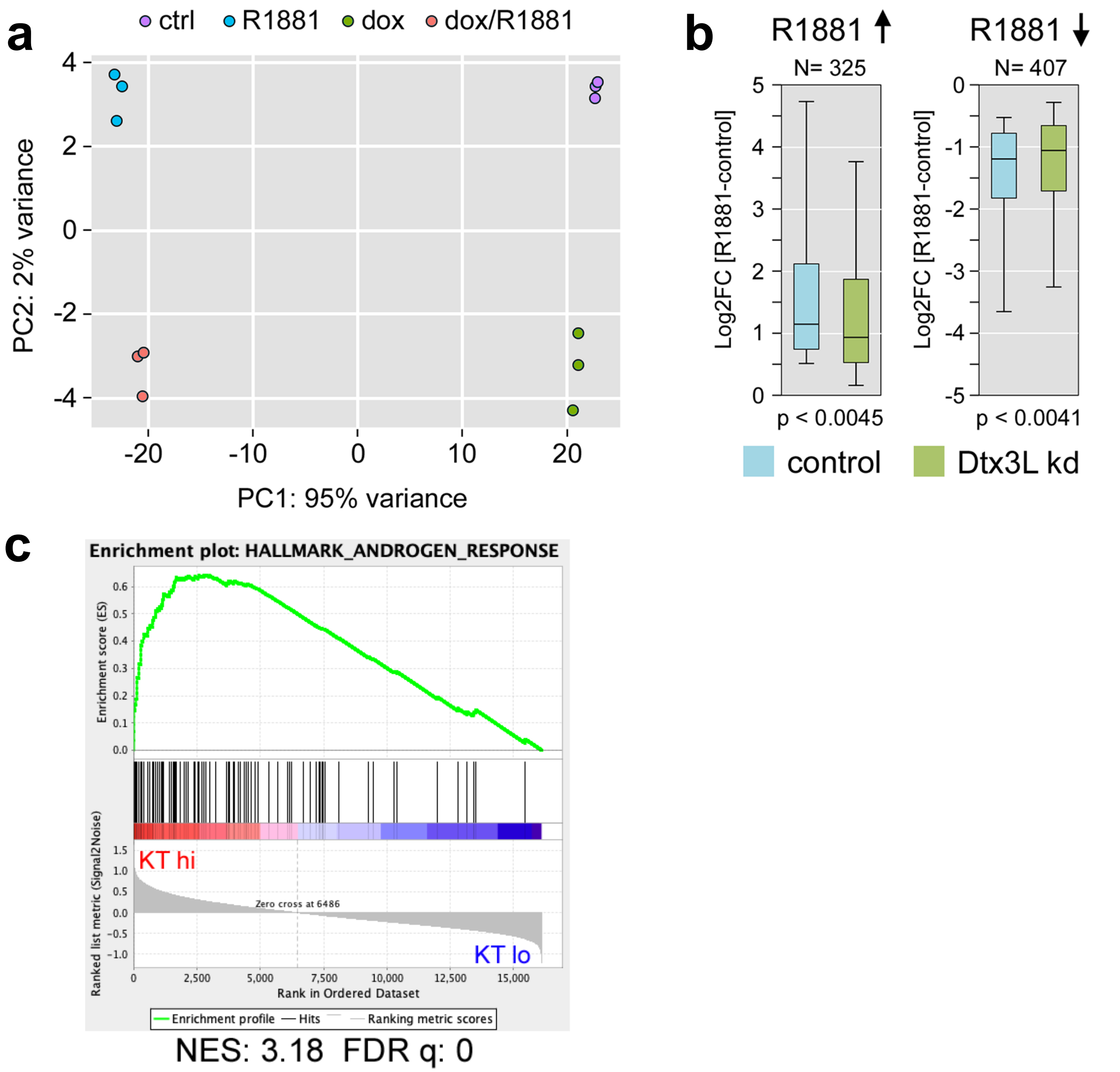

Supplementary Fig. 23. RNA-seq analysis of Dtx3L knockdown and R1881 treatment in VCaP cells.

(a) Principal component analysis (PCA) of RNA-seq data from VCaP cells +/- R1881 for 24 hrs, and +/- Doxycycline to induce the shRNA to Dtx3L (knock-down).

(b) Comparison of the $\log_2$-fold effect of R1881 treatment on activated (upward arrow) or repressed (downward arrow) genes without (blue) or with (green) Dtx3L knockdown. The data are shown as the median, 25th and 75th percentiles (box) and 5th and 95th percentiles (whiskers). p-values by Student's t test. Source data are provided as a Source data file.

(c) GSEA of RNA-seq data from TCGA PrAD data comparing top (KT hi) and bottom (KT lo) deciles by KLK3 + TMPRSS2 expression.

Supplementary Table 1. Mass spectra analyses of AR ADP-ribosylation.

AR peptide sequence	Modifications	XCorr	z	MH+ [Da]	Predicted MH+ [Da]	Mass Deviation (ppm)	Assignment[1]	Domain
RGPTGYLVLDEEQQPSQPQSALECHPER	C24(ADP-Ribosyl)	4.69	4	3705.5558	3705.5667	-2.9	C125	NTD
GDCMYAPLLGVPPAVRPTPCAPLAECK	C20(ADP-Ribosyl)	2.87	3	3424.4542	3424.4636	-2.7	C284	NTD
GLEGESLGCSGSAAAGSSGTLELPSTLSLYK	C9(ADP-Ribosyl)	2.93	3	3483.4856	3483.4909	-1.5	C327	NTD
LENPLDYGSAWAAAAAQCR	C18(ADP-Ribosyl)	3.63	3	2547.9957	2548.0000	-1.7	C406	NTD
GCVPEPGAAVAASK	C2(phosphoRibosyl)	2.25	2	1468.6393	1468.6389	0.3	C131	NTD
AECKGSLL	C3(phosphoRibosyl)	1.88	2	1032.4323	1032.4319	0.4	C290	NTD
AAAAAQCRY	C7(phosphoRibosyl)	2.43	2	1136.4444	1136.4442	0.2	C406	NTD
VPYPSPTCVK	C8(phosphoRibosyl)	2.47	2	1302.5688	1302.5687	0.1	C519	NTD
YLCASR	C3(phosphoRibosyl)	1.07	2	924.3531	924.3533	-0.2	C596	DBD
NDCTIDKFR	C3(phosphoRibosyl)	1.91	2	1323.5291	1323.5287	0.3	C602	DBD
CYEAGMTLGAR	C1(phosphoRibosyl)	1.24	2	1383.5324	1383.5320	0.3	C620	DBD
CQPIFLNVLE	C1(phosphoRibosyl)	2.01	2	1387.6219	1387.6215	0.3	C670	LBD

[1]ADP-ribosylated Cysteines in green. The C284(ADP-Ribosyl) peptide was carbamideomethyl modifed at C3 and C26.

Supplementary Table 2. Androgen-regulated genes in human prostate cancer affected by Dtx3L depletion in VCaP cells

TCGA PrAd top vs bottom 10% by Klk3 Tmprss2:			Dtx3L kd	
			with or without R1881	
p < e-7, +/- 1.0 log2-fold difference				
top 10% - bottom 10%:	> +1 log2	434	> +0.5 lfc	412
	< -1 log2	989	< -0.5 lfc	658

Dtx3L kd	Increased expression		Decreased expression	
TCGA PrAd	KT hi	KT lo	KT hi	KT lo
# genes	16	12	38	70
	CACNG4	ALDH1A1	ABCC4	ABTB2
	CHRNA5	ATP1B1	BBS4	AKR1C3
	FABP5	C12orf75	C1orf115	ALOX15
	GALNT7	CA2	C2orf72	ANO1
	HEBP2	CDCA7	CACNA1G	ANO6
	HIST1H2BE	LAMA3	CCDC108	ATP2B4
	HMGCS2	MMD	CECR6	C1R
	LENG9	QSOX1	COLEC12	C20orf194
	LPAR3	RERG	CPLX3	CA12
	MGST1	SPOCK2	DNAH5	CACNA1C
	PSAT1	ST3GAL1	DNAH9	CAMK2N1
	SLC16A1	ZFP36L1	ELFN2	CCDC88A
	SMS		FAM13C	CLIP3
	STRA13		FAM3B	COL16A1
	TBC1D4		FXYD3	COL27A1
	TMEFF2		GCNT2	CRABP2
			GDF15	CRIP2
			HIST1H2AC	DCLK1
			KLK3	DDIT4
			LCP1	DLG4
			LIPH	F2R
			LMAN1L	FHOD3
			MESP1	FMN2
			MYBPC1	GAB2
			NAAA	GDA
			NWD1	GPR153
			P4HB	GPR85
			PLA2G4F	IL17RD
			RAB27A	LAMC2
			REPS2	LMCD1
			SLC10A5	LRP1
			SLC23A1	LRRC43
			SLC39A10	MAP1A
			SLC4A4	MBNL2
			TMPRSS2	MCAM
			TRPM8	MCC
			TSPAN1	MGLL
			TTC39A	MYOF
				NDRG4
				NEURL3
				NFASC
				NID1
				NLRP1
				NPR1
				OGDHL
				PALLD
				PLEKHA2
				PPP1R12B
				PPP1R3C
				RARG
				RASSF2
				RASSF5
				RIMBP2
				SAMD4A
				SAMD9
				SHH
				SLC16A3
				SLC2A6
				SLFN5
				SORL1
				SOSTDC1
				SP6
				ST5
				STEAP3
				TMEM173
				TPST1
				TRNP1
				VTN
				WDR66
				ZFHX4

Supplementary Table 3. Sequences of oligonucleotides

shGFP insert sequence	CCGGTACAACAGCCACAACGTCTATCTCGAGATAGACGTTGTGGCTGTTGTATTTTT
shParp7 insert sequence	CCGGGAAGGCAAGCTACTCTCATAACTCGAGTTATGAGAGTAGCTTGCCTTCTTTTT
Tet-shDtx3L insert sequence	CCGGCGCGTATTGGAGTCTCAGTCTCGAGACTGAGACTCCAATACGCGTTTTT
Parp7 cDNA primer#1	TCTCAGGAGCACTTGGAAAGA
Parp7 cDNA primer#2	TCAGCCTTCGTAGTTGGTCA
Parp7 ChIP primer#1	ACAAGGCCCACGAAATAGTC
Parp7 ChIP primer#2	CACCCTGTGAGGAAGCAAAC
Mybpc1 cDNA primer#1	GTCGCTCTCACATGGACTCC
Mybpc1 cDNA primer#2	AATGGTGGCACTGGTTCGA
Mybpc1 ChIP site 1 primer#1	GATGAAGAAAGCTGCCCACG
Mybpc1 ChIP site 1 primer#2	ATAGTTGACTGGCAGGCTGT
Mybpc1 ChIP site 2 primer#1	GTTACTGCCACTGAGCCACT
Mybpc1 ChIP site 2 primer#2	AGCAGAATTTGGGAGGAGACA
CHIP Intergenic site Primer#1	CACCTGCACCTGCTTGTTT
CHIP Intergenic site Primer#2	CCAAGATCACACCACTGCAC
KLK3 cDNA primer#1	TGGTGCATTACCGGAAGTGGATCA
KLK3 cDNA primer#2	GCTTGAGTCTTGGCCTGGTCATTTC
MESP1 cDNA primer#1	CAGATGCAGACACGGACGCA
MESP1 cDNA primer#2	GGCATCCAGGTCTCCAACAGA
ABCC4 cDNA primer#1	GGCAGTGACGCTGTATGG
ABCC4 cDNA primer#2	CGCCAGGTCTGACAGTAAAG
Parp9 KO (sense) sg-Parp9-1	CTAGGATAGAGTTATCAGTC
Parp9 KO (sense) sg-Parp9-2	GAGATAGCTGTCACGGGAGC
Parp9 KO screening primer#1	TGCTGTGGTGAATGCAGCCAA
Parp9 KO screening primer#2	AGGCTGGAATTGCTACTGTCTT
Parp1C-activating pair oligo#1	CCGAATCAGTCCGACGACGCATCAGCAC
Parp1C-activating pair oligo#2	GTGCTGATGCGTCGTCGGACTGATTCGG
