# Supplementary material for: Androgen signaling uses a writer and a reader of ADP-ribosylation to regulate protein complex assembly
Source: Nat Commun. 2021 May 11;12:2705. doi: 10.1038/s41467-021-23055-6 (PMC8113490; doi:10.1038/s41467-021-23055-6)
Supplement: Supplementary file 3 — Description of Additional Supplementary Files [file 41467_2021_23055_MOESM3_ESM.docx]

File Name: Supplementary Data 1

Description: Dtx3L and androgen signaling in VCaP cells

GSE133876: [<https://www.ncbi.nlm.nih.gov/geo/query/acc.cgi?acc=GSE133876>]

Summary: Gene expression in VCaP prostate cancer cells treated with or without androgen was analyzed. VCaP cells containing a dox-inducible shRNA against Dtx3L were compared with and without dox induction (Dtx3L knockdown), in the presence or absence of androgen.

Overall design: RNA was extracted from VCaP cells with a Dox-inducible Dtx3L shRNA, either untreated or treated with androgen for 24 hours (2 nM, R1881). Two conditions were sequenced with 3 replicates for each condition. Data from dox-treated (Dtx3L knockdown) VCaP cells (+/- R1881) was analyzed together with data from control (no dox) VCaP cells (+/- R1881).
