# Supplementary material for: Androgen signaling uses a writer and a reader of ADP-ribosylation to regulate protein complex assembly
Source: Nat Commun. 2021 May 11;12:2705. doi: 10.1038/s41467-021-23055-6 (PMC8113490; doi:10.1038/s41467-021-23055-6)
Supplement: Supplementary file 5 — Reporting Summary [file 41467_2021_23055_MOESM5_ESM.pdf]

nature research

Corresponding author(s): Bryce Paschal

Last updated by author(s): 04-07-2021

Reporting Summary

Nature Research wishes to improve the reproducibility of the work that we publish. This form provides structure for consistency and transparency in reporting. For further information on Nature Research policies, see our Editorial Policies and the Editorial Policy Checklist.

Statistics

For all statistical analyses, confirm that the following items are present in the figure legend, table legend, main text, or Methods section.

n/a	Confirmed	
☐	☒	The exact sample size (*n*) for each experimental group/condition, given as a discrete number and unit of measurement
☐	☒	A statement on whether measurements were taken from distinct samples or whether the same sample was measured repeatedly
☐	☒	The statistical test(s) used AND whether they are one- or two-sided<br>*Only common tests should be described solely by name; describe more complex techniques in the Methods section.*
☒	☐	A description of all covariates tested
☒	☐	A description of any assumptions or corrections, such as tests of normality and adjustment for multiple comparisons
☐	☒	A full description of the statistical parameters including central tendency (e.g. means) or other basic estimates (e.g. regression coefficient) AND variation (e.g. standard deviation) or associated estimates of uncertainty (e.g. confidence intervals)
☐	☒	For null hypothesis testing, the test statistic (e.g. *F*, *t*, *r*) with confidence intervals, effect sizes, degrees of freedom and *P* value noted<br>*Give P values as exact values whenever suitable.*
☒	☐	For Bayesian analysis, information on the choice of priors and Markov chain Monte Carlo settings
☒	☐	For hierarchical and complex designs, identification of the appropriate level for tests and full reporting of outcomes
☒	☐	Estimates of effect sizes (e.g. Cohen's *d*, Pearson's *r*), indicating how they were calculated

Our web collection on statistics for biologists contains articles on many of the points above.

Software and code

Policy information about availability of computer code

Data collection	Mass Spectrometry data was collected using multiple instruments in our core facility at UVA. The instrument software used was Foundation 2.1, Xcalibur 2.3, LC Devices 3.1, and Orbitrap MS 2.3. RNA-seq data was collected by HudsonAlpha on an Ilumina HiSeq 2500 running HCS version 2.2.68.. Fluorescence microscopy data was collected using a Nikon Eclipse Ni-U microscope and DS-Qi1Mc camera running NIS-Elements software version 4.13.
Data analysis	Mass Spectrometry data was analyzed using Sequest, Proteome Discoverer 2.2, and Scaffold 4.10.0. RNA-seq data was analyzed using Salmon TPM counts and DEseq2 on the Galaxy Server. Fluorescence microscopy data was analyzed using ImageJ version 1.52i (NIH) and Prism8 for MacOS (GraphPad).

For manuscripts utilizing custom algorithms or software that are central to the research but not yet described in published literature, software must be made available to editors and reviewers. We strongly encourage code deposition in a community repository (e.g. GitHub). See the Nature Research guidelines for submitting code & software for further information.

Data

Policy information about availability of data

All manuscripts must include a data availability statement. This statement should provide the following information, where applicable:

- Accession codes, unique identifiers, or web links for publicly available datasets
- A list of figures that have associated raw data
- A description of any restrictions on data availability

We will make freely available any datasets necessary to interpret and replicate the methods and findings reported in this article. Source data for all figures is contained in the Source Data file.
RNA-seq data generated in our studies is publically available at GEO.

GSE120660 [https://www.ncbi.nlm.nih.gov/geo/query/acc.cgi?acc=GSE120660] (Androgen effect on PC3-AR and VCaP cells)
GSE133876 [https://www.ncbi.nlm.nih.gov/geo/query/acc.cgi?acc=GSE133876] (Dtx3L and androgen effect in VCaP cells)

AR ChIP-seq data previously published31,32 is available at GEO. GSE28126 [https://www.ncbi.nlm.nih.gov/geo/query/acc.cgi?acc=GSE28126] (Androgen effect on AR ChIP-seq in VCaP cells). GSE54202 [https://www.ncbi.nlm.nih.gov/geo/query/acc.cgi?acc=GSE54202] (Androgen effect on AR ChIP-seq in PC3-AR cells).

The mass spectrometry proteomics data have been deposited to the ProteomeXchange Consortium via the PRIDE [https://www.ebi.ac.uk/pride/archive/] partner repository with the dataset identified PXD018811 [https://www.ebi.ac.uk/pride/archive/projects/PXD018811/private] (AR ADP-ribosylation sites) and PXD025195 [https://www.ebi.ac.uk/pride/archive/projects/PXD025195/private] (Parp7 ADP-ribosylation sites).

Protein structure data from other groups used were 5AIL [https://www.rcsb.org/structure/5AIL] (Parp9 MD2), 2BFQ [https://www.rcsb.org/structure/2BFQ] (Af1521 structure) and 3VFQ [https://www.rcsb.org/structure/3VFQ] (Parp14 MD2).

Field-specific reporting

Please select the one below that is the best fit for your research. If you are not sure, read the appropriate sections before making your selection.

☒ Life sciences ☐ Behavioural & social sciences ☐ Ecological, evolutionary & environmental sciences

For a reference copy of the document with all sections, see nature.com/documents/nr-reporting-summary-flat.pdf

Life sciences study design

All studies must disclose on these points even when the disclosure is negative.

Sample size	For Statistical analysis, gene expression experiments were performed in triplicates. To account for biological variation, microscopy experiments utilized at least 64 cells per condition.
Data exclusions	There were no data exclusions other than mitotic cells.
Replication	All attempts at replication were successful. (For biochemical observations: n=2; For mass spectrometry and RNA-seq, n=1).
Randomization	No randomization was performed or was relevant to the study because there was no population-based data analysis.
Blinding	Blinding was not used in the microscopy studies because fields of cells were randomly selected and every cell was counted, unless it was a mitotic cell.

Reporting for specific materials, systems and methods

We require information from authors about some types of materials, experimental systems and methods used in many studies. Here, indicate whether each material, system or method listed is relevant to your study. If you are not sure if a list item applies to your research, read the appropriate section before selecting a response.

Materials & experimental systems

n/a	Involved in the study	
☐	☒	Antibodies
☐	☒	Eukaryotic cell lines
☒	☐	Palaeontology and archaeology
☒	☐	Animals and other organisms
☒	☐	Human research participants
☒	☐	Clinical data
☒	☐	Dual use research of concern

Methods

n/a	Involved in the study	
☒	☐	ChIP-seq
☒	☐	Flow cytometry
☒	☐	MRI-based neuroimaging

Antibodies

Antibodies used	Lab produced antibodies: AR (656-669AA) rabbit polyclonal (Western blot, WB: 1μg/ml), Parp9 Catalytic Domain rabbit polyclonal (WB: 1μg/ml), Dtx3L Catalytic Domain rabbit polyclonal (WB: 0.2μg/ml), Parp7 rabbit polyclonal (119-132AA) (WB: 1μg/ml), and AR phospho-site antibodies (WB: 1-1,000-2,000). Commercial antibodies: flag epitope (WB: 0.75 μg/ml, M2, Sigma-Aldrich, A2220); AviTag rabbit polyclonal (WB: 1μg/ml, GenScript, A00674-100); Parp1 rabbit polyclonal (WB: 1:1,000, abCam ab32138); alpha-tubulin mouse monoclonal (WB: 1:10,000, Sigma, T 9028); HA epitope mouse monoclonal (WB and immunofluorescence microscopy, IF: 1-1,000, Covance, A488-101L); HA epitope rabbit monoclonal (IF: 1:500, Cell Signaling Technology, 3724S); Alexa Fluor 680 donkey anti-Rabbit IgG(H+L) (WB: 1:20,000, Invitrogen A10043), IRDye800-Conjugated anti-Mouse IgG(H&L) (WB: 1:20,000, Rockland 610-132-121), Cy™3 AffiniPure Donkey Anti-Rabbit IgG (H+L) (IF: 1:400, Jackson Immuno Research Laboratories, Inc. 711-165-152) and Alexa Fluor® 488 AffiniPure Donkey Anti-Mouse IgG (H+L) (IF: 1:200, Jackson Immuno Research Laboratories, Inc. 715-545-150),

	and Neutravidin-DyLight-800 (WB: 1:20,000, Pierce 22853).
Validation	Lab anti-AR, AR phopho-sites, Dtx3L, Parp9 and Parp7 were published (PMC1899975, Yang et al, Mol Cell Biol. 2007, 27(9):3390-404; PMC7916378, Kamata et al, Cells. 2021, 10(2):363). Commercial mouse anti-Tyrosine Tubulin is immunospecific for tubulin as determined by indirect immunofluorescence staining of cultured chicken fibroblasts; rabbit anti-Parp1 is KO validated. Epitope tag antibodies (M2, HA, MBP, and Avi tag) were validated by immunoblotting with fusion proteins. More information could be obtained in their websites.

Eukaryotic cell lines

Policy information about cell lines

Cell line source(s)	VCaP prostate cells (ATCC CRL-2876), HEK293T cells (ATCC CRL-3216), PC3 (ATCC CRL-1435) were from ATCC. PC3M (RRID:CVCL_9555) was a gift from Michael Weber Lab. PC3-AR, PC3M-HA-AR and other stable cell lines in the paper are derivatives of PC3 and PC3M.
Authentication	Cell lines were not authenticated after receipt from ATCC or gifted from Michael Weber Lab.
Mycoplasma contamination	Cell lines were mycoplasma-free when purchased from ATCC or from Weber Lab, but were not re-tested during the study.
Commonly misidentified lines (See ICLAC register)	None of the cell lines used are on the mis-identified cell line list (March 2020 release).
